# Breast Cancer Diagnostic Delays Among Young Mexican Women Are Associated With a Lack of Suspicion by Health Care Providers at First Presentation

**DOI:** 10.1200/JGO.19.00093

**Published:** 2019-07-23

**Authors:** Karla Unger-Saldaña, Kenneth Fitch-Picos, Cynthia Villarreal-Garza

**Affiliations:** ^1^ Instituto Nacional de Cancerología, Mexico City, Mexico; ^2^ Breast Cancer Center, Hospital Zambrano Hellion, Tecnologico de Monterrey, Monterrey, Mexico

## Abstract

**PURPOSE:**

There is insufficient evidence in the literature regarding the association between young age and diagnostic delay of breast cancer (BC). This study aimed to determine whether young age increases the risk of diagnostic delays among patients with BC and also to identify the mechanisms through which young age affects diagnostic delay.

**CONTEXT**

**Key Objective**
This study aimed to determine if young age increases the risk of diagnostic delays among patients with breast cancer and also to identify the mechanisms through which young age affects diagnostic delay. A cross-sectional study of 592 patients with symptomatic breast cancer treated at two of the largest public cancer hospitals in Mexico City was performed.
**Knowledge Generated**
Participants 40 years of age or younger had significantly longer diagnostic intervals and presented with more advanced cancers than did their older counterparts. Young age increased the risk of diagnostic delays through an increased risk of lack of cancer suspicion at the first health care service consulted.
**Relevance**
It is of the uttermost importance to improve cancer awareness among primary health care providers working in the public system so that they avoid discarding a cancer diagnosis solely on the basis of the patient’s age.

**PATIENTS AND METHODS:**

This was a cross-sectional study of 592 patients with symptomatic BC treated at two of the largest public cancer hospitals in Mexico City available for the uninsured and those covered by Seguro Popular. A validated questionnaire was administered via face-to-face interviews with the patients, and their medical files were reviewed. Path analyses, using multivariable logistic regression models, were conducted to assess the relationship between age and diagnostic delay, as well as the role of potential confounders.

**RESULTS:**

Younger participants (40 years of age or younger) had significantly longer diagnostic intervals and presented with more advanced cancer stage than did their older counterparts. Younger participants more often sought initial health care in private services led by gynecologists, more frequently experienced a lack of cancer suspicion by the first physician they consulted, used a higher number of different health services, and had more medical consultations before arrival to a cancer care center. Younger age was significantly associated with longer diagnostic delays after controlling for education, occupation, lack of health insurance, history of benign breast conditions, type of first health service used, specialty of the first physician consulted, first symptom presented, and benign interpretation of the first breast image study.

**CONCLUSION:**

Young age increased the risk of diagnostic delays, which seems to be a result of an increased risk of lack of cancer suspicion at the first health care service consulted.

## INTRODUCTION

Breast cancer (BC) is the most common cancer affecting women worldwide.^1^ Mortality as a result of BC is higher in low- and middle-income countries (LMIC) than in high-income countries.^1^ This has been shown to be a consequence of late-stage presentation and limited access to standard treatment options.^2^ There is evidence that delays between symptom discovery and the start of cancer treatment negatively affect clinical stage and, thus, survival.^3,4^

In LMIC, a large proportion of BC cases presents in women younger than 50 years of age,^5,6^ with worse survival rates in comparison with older women.^7,8^ Having a BC diagnosis has a major impact on young women’s lives because it affects their integral development in a stage of life when they are commonly consolidating financially and professionally, with many having young children or starting a family.^9^

Poor outcomes among young women with BC have been explained mainly by biologic mechanisms. Young women have higher rates of negative estrogen receptor activity, resistance to hormone therapy, Ki-67 expression, and cancer recurrence.^10,11^ In addition, worse outcomes reported for younger women could also be explained by diagnosis in more advanced stages because of delays in receiving a timely and appropriate work-up.^12-14^

Studies analyzing the relationship between young age and diagnostic delay have had contradictory findings.^15-18^ These studies are difficult to compare because they have different operational definitions for young age and for diagnostic interval. Thus, there is insufficient evidence in the literature regarding the association between young age and diagnostic delay. This study aimed (1) to determine if young age increases the risk of diagnostic delays among patients with BC treated at two of the main public hospitals available for the uninsured in Mexico City and (2) to identify the mechanisms through which young age affects diagnostic delay.

## PATIENTS AND METHODS

### Design

We conducted a cross-sectional study including patients with BC who were first diagnosed between June 2016 and May 2017 in two of the largest public hospitals available for the uninsured in Mexico City: the Mexican National Cancer Institute and the General Hospital of Mexico Eduardo Liceaga. The study protocol was approved by the participating hospitals’ research boards. Informed consent was obtained from all participants.

### Setting

The Mexican National Cancer Institute and the General Hospital of Mexico Eduardo Liceaga fall under the purview of the Ministry of Health, which offers health services in exchange of income-related user fees for the uninsured and without cost for those covered by Seguro Popular, which is a federal program that permits its affiliates to benefit from an explicit list of health interventions. In addition, the Fund for Protection Against Catastrophic Health Expenditures covers high-cost interventions, such as BC treatment, for both the uninsured and those covered by Seguro Popular. According to the most recently conducted National Survey of Health and Nutrition (2016), 43.5% of the Mexican population is covered by Seguro Popular, and 13.4% remain uninsured.^19^

### Participants

**FIG 1:**
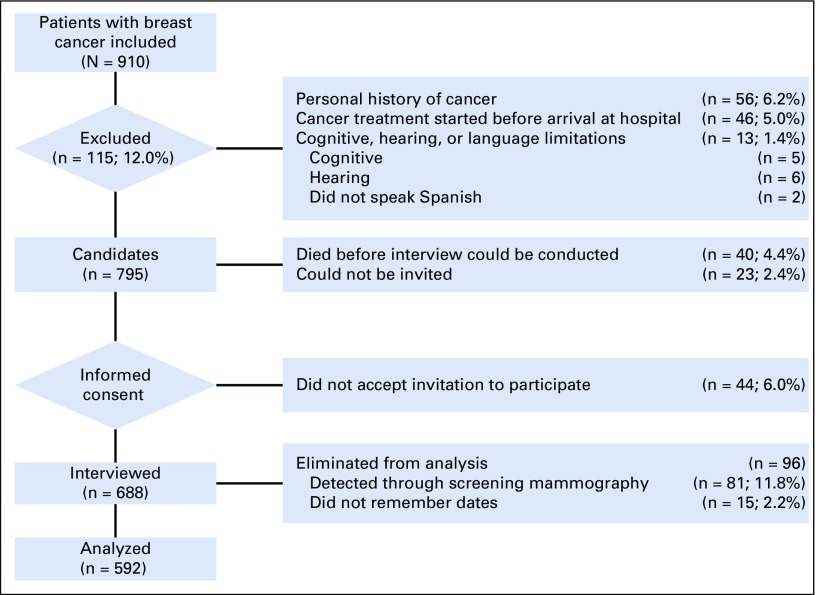
Participant inclusion, exclusion, and elimination criteria.

Figure 1 presents the participants’ inclusion, exclusion, and elimination criteria. Overall, 910 patients first sought care at the breast tumor departments of the participating hospitals over the study period. Exclusion criteria included patients who (1) had a personal history of cancer (56 of 910; 6.2%); (2) began systemic cancer treatment before arrival at the cancer institution (46 of 910; 5.0%); (3) could not participate in the interview for various reasons (ie, cognitive disability, hearing impairment, did not speak Spanish (13 of 910; 1.4%); (4) died shortly after their arrival at the cancer institution before an interview was conducted (40 of 910; 4.4%); and (5) could not be located in order to invite them to the study (23 of 910 patients; 2.5%). Seven hundred thirty-two (80.4%) of 910 patients who fulfilled the inclusion criteria were invited to the study, and of those, 44 (6.0%) of 732 were not willing to participate. Six hundred eighty-eight patients (93.9%) of 732 were interviewed and their medical records were reviewed. Finally, 15 (2.2%) of 688 participants were eliminated from analysis because they could not recall the dates required to estimate the diagnostic interval and 81 (11.8%) of 688 patients were eliminated because their BC was detected by screening. This last criterion was included to control for confounding because screening is not recommended among women younger than 40 years of age; thus, the vast majority of women screened would have been 40 years of age or older. Abnormal mammography screening results would result in a different diagnostic pathway; therefore, to make the analysis by age group more comparable, we decided to analyze only women with symptomatic presentations, which comprised 88.2% of the participants.

### Measure of the Diagnostic Interval

The diagnostic interval was defined as the time from the first medical consultation that the patient sought for her breast symptoms to the first report of histopathologic confirmation of BC. This definition is in line with the recommendations of the Aarhus Statement on the design and reporting of studies on early cancer diagnosis.^20^ For the logistic regression analyses, diagnostic delay was defined as more than 90 days between first consultation and diagnostic confirmation. Although there is no consensus in the literature, the most common threshold used to consider diagnosis delay in previous studies has been 1 month. However, we decided not to use the 1-month threshold in the current study because only 26% of our participants had diagnostic intervals of 30 days or less. The median diagnosis interval was 63 days; therefore, we decided to use the 3-month threshold that corresponded to the 60th percentile in our data.

### Data Collection

A validated questionnaire was used to retrieve the date of the first medical consultation that the patient received after her discovery of breast symptoms.^21^ We also retrieved information on the sociodemographic characteristics of the participants, and patient perceptions and experiences with the medical services that were used before arrival at the cancer hospital. The patients were interviewed face to face at the participating hospitals by psychology and medical students who were trained to standardize the questionnaire application. To minimize the probability of recall bias, study participants were asked to remember dates using the aid of a calendar. Data regarding each patient’s clinical characteristics and date of diagnostic confirmation were extracted from the patient’s hospital records.

### Statistical Analysis

**FIG 2:**
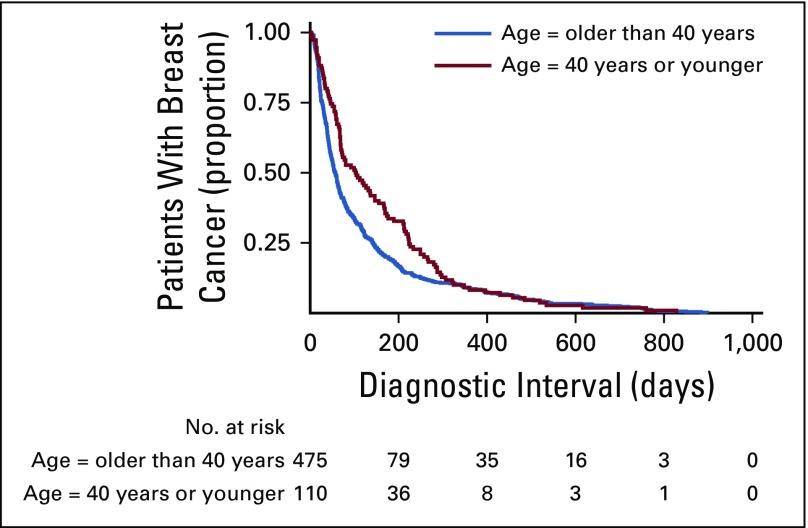
Kaplan-Meier curves of the diagnostic interval stratified by patient age. These curves show a significantly longer diagnostic interval among women 40 years of age and younger in comparison with their older counterparts.

**FIG 3:**
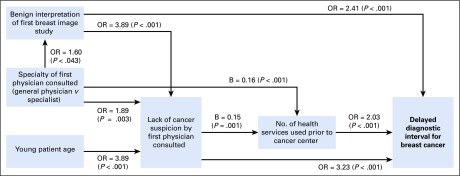
Mechanisms through which young age influences diagnostic delay. This path diagram represents causal relationships between variables. Where there are no arrows between variables, no association was found. The reported regression coefficients or odds ratios (ORs) on each arrow were adjusted using multivariable analyses that included the variables that appear on the diagram to the left of each dependent variable. The ORs were estimated using logistic regression analysis when the dependent variable was categorical, and the B coefficients were estimated using lineal regression for continuous dependent variables. For example, for the analysis of the diagnostic interval, which was a binary response variable, the OR of 3.23 of lack of cancer suspicion by the first doctor consulted is the adjusted OR obtained by a multivariable logistic regression where all the variables in the diagram were included as controls. As can be seen, young patient age is not directly associated with diagnostic delay, but delays occur because of a lack of cancer suspicion by the first doctor consulted.

Descriptive statistics were estimated for all variables. The χ^2^ test was used to assess differences in descriptive variables by age group (≤ 40 *v* > 40 years; Tables 1 and 2). Kaplan-Meier curves were generated to examine the association between the diagnostic interval and patient age (Fig 2). Diagnostic confirmation was defined as the censoring event, and a Cox regression model was built to identify significant differences in interval length between the two different groups. Logistic regression analyses were performed between age and diagnostic delay, as well as potential confounders of this relationship (patient education, occupation, marital status, lack of health insurance, family income, hospital of cancer care, history of benign breast conditions, type of first health service used, specialty of first physician consulted, number of different health services used, benign interpretation of first breast imaging study, and lack of BC suspicion after first medical consultation). Variables with odds ratio *P* values of < 0.1 were included in the multivariable logistic regression analyses to adjust for potential confusion. All models included the age variable, and adjustment variables were gradually incorporated to test different models. Table 3 lists the most relevant models that resulted from our analyses. Finally, a path analysis was undertaken to learn how much of the relationship between age and diagnostic delay is accounted for by intervening or mediating factors. In path analysis, the dependent variable in one equation may serve as the control in another equation without statistically complicating matters.^22^ The inclusion and order of variables in our path analysis was based on previous work—a conceptual model that resulted from a qualitative study of help-seeking behavior of women with BC, and a path diagram that resulted from a quantitative study on factors associated with BC health system delay.^23,24^ Each equation was estimated by taking into account the nature of the dependent variable: logistic regression for binary responses and linear multivariable regressions for continuous responses. The results of the path analysis are represented in a path diagram in which straight unidirectional arrows represent causal relationships (Fig 3). The odds ratio (for dependent binary variables) or B coefficient (for dependent continuous variables) presented above each arrow is the adjusted estimator that accounts for the relationship between the two variables connected by the arrow.

**TABLE 1:**
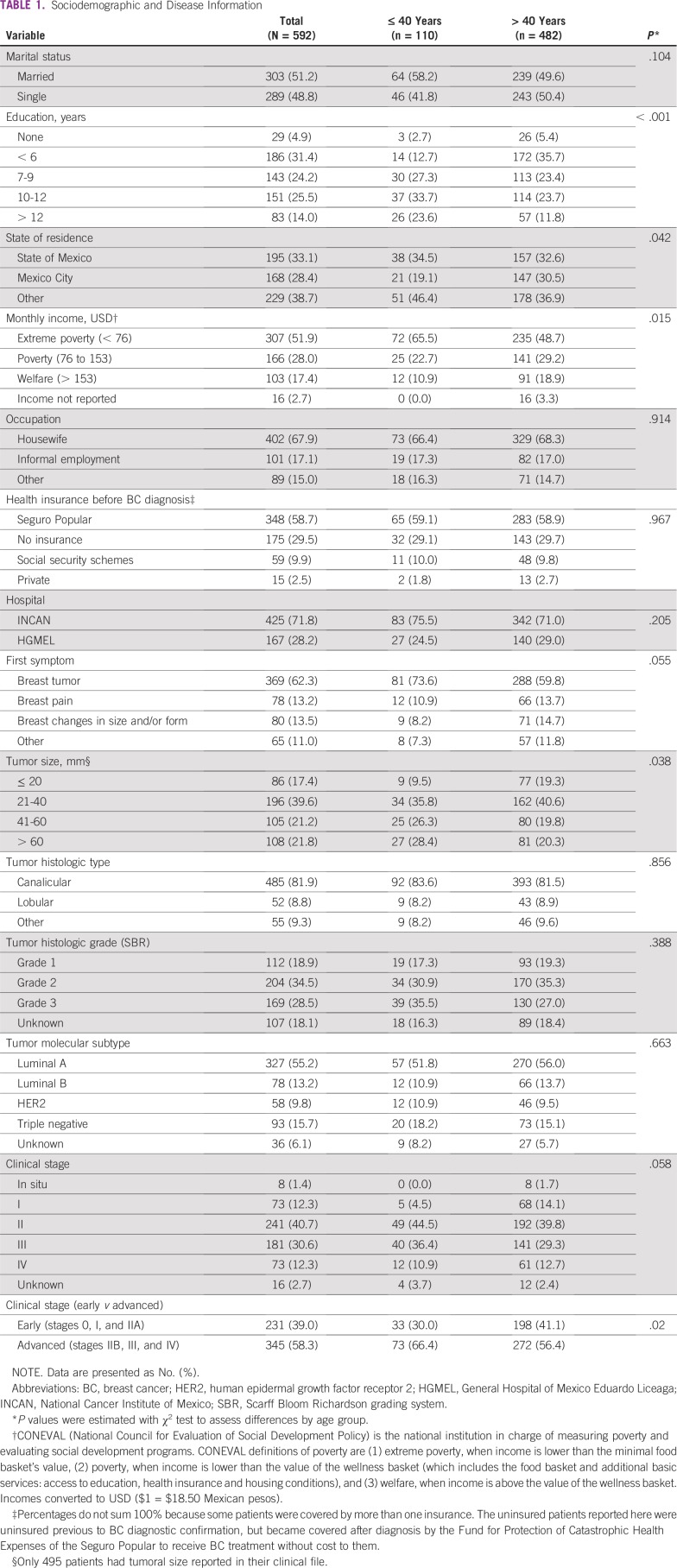
Sociodemographic and Disease Information

**TABLE 2:**
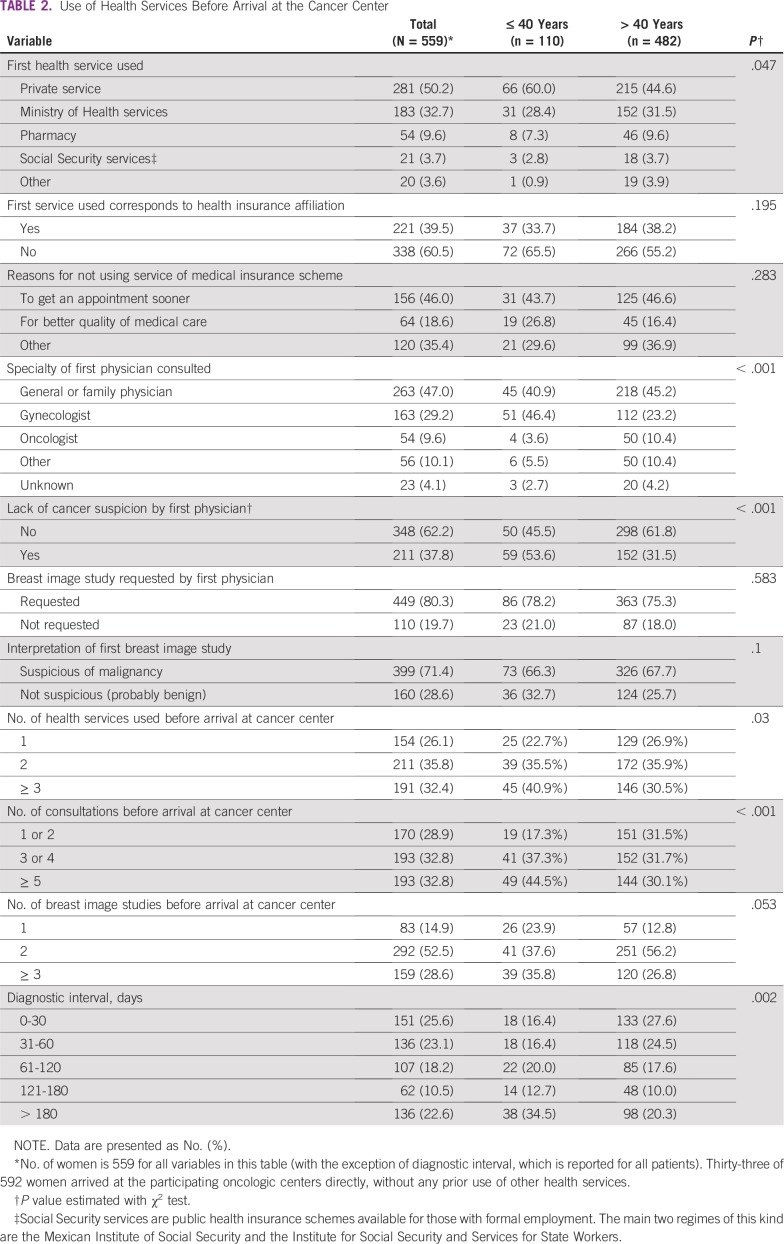
Use of Health Services Before Arrival at the Cancer Center

**TABLE 3:**
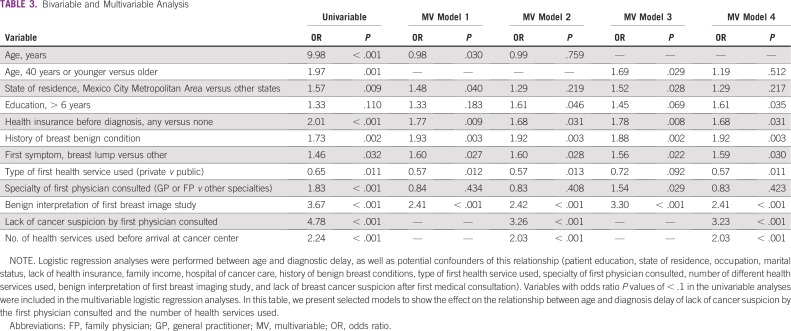
Bivariable and Multivariable Analysis

## RESULTS

We report data on 592 of 910 patients who fulfilled the inclusion criteria. Sociodemographic and clinical characteristics are listed in Table 1. The average age of presentation was 51.35 years (SD, 12.53 years; range, 23 to 91 years). One hundred ten (18.6%) of 592 patients were 40 years of age or younger. The majority of our participants had low levels of education; approximately 55% completed up to 9 years and 25% up to 12 years of school. A total of 67.9% of our participants were unemployed, 80% reported household incomes below the national line of poverty, and 30% lacked any form of health insurance before arrival at the participating cancer centers. Patients 40 years of age and younger tended to have higher education levels, originate more often from states other than Mexico City, and report lower household incomes. The majority of our participants presented in advanced stages. Young patients presented with larger tumors and more advanced disease (Table 1).

Younger participants had significantly longer diagnostic intervals than did their older counterparts, with a median time of 103 days (25th percentile, 46 days; 75th percentile, 224 days) for patients 40 years of age and younger, in comparison with a median of 57 days (25th percentile, 27 days; 75th percentile, 146 days) for patients older than 40 years of age (Fig 2 and Table 2). Younger participants more often sought initial health care in private services led by gynecologists, more frequently experienced a lack of cancer suspicion by the first physician they consulted, used a higher number of different health services, and had more medical consultations before arrival at a cancer care center (Table 2).

Table 3 lists the selected models of the multivariable analyses used to understand the relationship between age and diagnostic delay. Age was significantly associated with longer diagnostic delays after controlling for education, occupation, lack of health insurance, history of benign breast conditions, type of first health service used, specialty of the first physician consulted, first symptom presented, and benign interpretation of the first breast image study (models 1 and 3). Each additional year of a patient’s age reduced by 2% the odds of having diagnostic delays greater than 3 months. Being 40 years old or younger increased a woman’s odds of facing a diagnostic delay in 69% compared with older women. However, this significance was lost when we included in the model either one of the following variables, or the two of them together: lack of cancer suspicion by the first physician consulted and number of health services used before arrival at the cancer center (model 4). These results were consistent whether age was analyzed as a continuous variable or as a categorical variable with a cutoff value of 40 years. We included interaction terms in the model and found no interactions between young age and history of benign breast conditions, young age and first symptom, and young age and lack of cancer suspicion by the first physician consulted.

To better understand the relationship between age and the diagnostic interval, we undertook path analyses. In our sample, the most relevant determinants of diagnostic delay were diagnostic errors, whether lack of suspicion by the first consulted clinician or a wrongful benign interpretation of the first breast imaging study. Our results suggest that young patient age increases the risk of diagnostic delay through an increased risk of cancer misdiagnosis by the first consulted doctor (Fig 3). The lack of suspicion of cancer in the first medical consultation increases the risk of diagnostic delay directly and, also, through an increased use of different health services before arrival at the cancer center. The main variables that increase the risk of having a lack of cancer suspicion after the first medical consultation are young age, lack of specialty of the consulted doctor, and a benign interpretation of the first breast imaging study (which most likely reveals medical errors in image interpretation).

## DISCUSSION

Our findings show that in our sample, composed of patients with BC in the two main public cancer hospitals available for the uninsured or people covered by Seguro Popular in the Mexico City Metropolitan Area, young age increased the risk of diagnostic delays, which seems to be caused by an increased risk of lack of cancer suspicion at the first health care service consulted. Younger women suffered more diagnostic delays and presented with more advanced cancer stage than did their older counterparts. The association between lack of cancer suspicion and younger women was independent of the type of first health care service used and the specialty of the first consulted doctor, despite the fact that younger women more commonly first used private services and saw gynecologists instead of general physicians, in comparison with women older than 40 years. We now discuss these findings in comparison with previous findings reported in the literature and in the Mexican context.

Although there are several reports on the relationship between age and different time intervals of care, we found only four papers in which the diagnostic interval was analyzed in relation to young age. These report conflicting findings: two studies found an association between young age and diagnostic delay,^15,16^ whereas the other two did not.^17,18^ These were all large sample studies, with strong statistical analyses, performed in different countries: the United States of America,^16,17^ the United Kingdom,^15^ and China.^18^ Although none of these studies adhered to the diagnostic interval definition proposed in the Aarhus Statement,^20^ Stuver et al,^16^ Partridge et al,^17^ and Huo et al^18^ used comparable definitions: time from first symptom discovery to diagnostic confirmation (which includes the patient interval plus the diagnostic interval). The paper by Neal et al^15^ measured the referral interval (ie, the time between first contact with primary care and referral to a specialist). It is interesting to note that Neal et al^15^ also reported a lack of association between age and delay when analyzing the prehospital interval (equivalent to the definitions used by Stuver,^16^ Partridge,^17^ and Huo^18^), but they found a significant relationship between young age and both referral delay (time between first contact with primary care and referral to a specialist) and secondary care delay (time between first referral to a specialist and treatment start). As for the studies by Partridge et al^17^ and Huo et al,^18^ they both found significant crude associations between young age and the time interval they analyzed that then disappeared when controlling by symptom presentation, menstrual status,^18^ and history of benign breast conditions,^18^ which are all related to young age.

It has been hypothesized previously that the relationship between young age and delays in the health care provider and/or diagnostic intervals is caused by a greater difficulty to suspect BC in this age group.^15,25,26^ These difficulties in suspecting BC are present in both the patients themselves and the physicians they consult, because young women are at a lower risk of BC in comparison with their older counterparts and because of the common presence of benign breast conditions among younger women. Even though medical errors have been hypothesized as the reason for delay among younger women, this is the first study to explore the medical diagnostic impressions experienced by patients with symptomatic BC at their first medical consultation and their relation to delayed diagnosis. Our findings show a significant association between young age and diagnostic delay after controlling for symptom presentation and history of benign breast conditions, in contrast to the previously mentioned studies.^17,18^ However, the association disappeared when diagnostic errors and/or number of health services used were added to the model. These results, plus our path analyses findings, suggest that it is through diagnostic errors and an increased use of health services that young age influences diagnostic delay.

The relationship between young age and health care delays may well be different in diverse health system contexts. The lengths of the provider and diagnostic intervals heavily depend on the availability and quality of cancer care services. In the case of Mexico, the gate-keepers of the primary care public health services available for the uninsured and those covered by Seguro Popular are general practitioners who have in most cases not been given the necessary training to suspect BC early and to initiate the appropriate diagnostic work-up. In addition, there are no clear referral routes for expedited diagnostic evaluation of women with BC symptoms. The alternative is to use affordable private services, which are heterogeneous in quality and completely separate from the public system.

An additional result that deserves a comment is the higher risk of delay among women who had any kind of health insurance previous to their arrival at the cancer institution, in comparison with those who were uninsured. This finding seems paradoxical and we cannot fully explain it. It is possible that, because of their lack of insurance, the health care personnel that they consulted (either in public or private services) considered their cases in special ways and accelerated care for them. This has been reported in the literature as “the waiting time paradox”, which describes the inverse relationship sometimes found between delay and clinical stage.^27^

One last comment we want to make is in regard to tumor size at arrival at the cancer center, because this was less than 20 mm in 17.4% of cases despite the fact that patients discovered the symptoms themselves. We double-checked the medical files to confirm this result. In fact, it has been reported previously that the median tumor size of a self-detected BC is between 19 and 22 mm.^28^

One study limitation was that causality could not be established because of the study’s cross-sectional design. Another potential limitation is that recall bias could have affected the precision of the measurement of intervals (data regarding symptom discovery and first medical consultation) and other variables. Nevertheless, the instrument used in this study demonstrated good reliability for the estimation of intervals of care in a previous validation process,^21^ and memory bias was minimized by conducting the interviews as early in the diagnostic process as possible. The diagnostic impression that resulted from this first encounter was not obtained from the medical files but from the patients themselves. Even though a review of the medical files of primary care services would have been ideal to get the precise date of first medical consultation and the initial medical diagnostic impression, this was not possible because there are no electronic medical files in public primary care services and because patients use many different health services.

Finally, our results are generalizable only to patients treated at these two federal hospitals, which offer cancer care to uninsured patients and to those covered by Seguro Popular, and who reside mostly in Mexico City and the surrounding states. However, it should be taken into consideration that the uninsured and those covered by Seguro Popular account for approximately 57% of the Mexican population, so our results remain relevant for the largest proportion of patients in the Mexican health care system. It remains to be studied whether similar problems occur in other medical institutions, especially those available for the insured under social security schemes, and in other regions of the country.

Our findings reveal insufficiencies in the health system for the early diagnosis of patients with symptomatic cancer. BC control policy in the last decades in Mexico has prioritized mammography screening, with limited success. According to the last National Survey of Health, the national BC screening coverage is approximately 20%,^29^ and in our sample, 86% of patients with BC presented symptomatically. Symptomatic patients account for the vast majority of patients with BC, even in more developed countries with well-established screening programs. The current study, in concordance with previous studies in Mexico, shows the long diagnostic delays that patients with symptomatic BC face, which put them at risk of tumor progression and worse outcomes.^30,31^ According to WHO guidelines, strengthening effective early cancer diagnosis provides the foundation for comprehensive cancer control, and ensuring sufficient capacity for early diagnosis and treatment is critical before initiating screening services.^32^ For example, the reduction in mortality and incidence ratios by one half in the United States of America from 1950 to 1975, before the introduction of mammography screening, is hypothesized to be a consequence of general improvements in BC awareness, increased detection of palpable masses, and better diagnostics.^33^ That is, much can be done to improve BC survival in LMIC in the short term, by strengthening early diagnosis capacity and access to treatment.

We have shown that younger patients treated at facilities available for the uninsured and those covered by Seguro Popular are especially vulnerable to diagnostic delays, and that this is apparently mainly because of a lack of suspicion of cancer among primary care physicians. It is of the utmost importance to improve cancer awareness among primary health care providers working in the public system so that they avoid discarding a cancer diagnosis solely on the basis of the patient’s age. In addition, there is a need for more clear and expeditious routes for the diagnostic assessment of patients in whom cancer is suspected and for treatment initiation among those with a confirmed cancer diagnosis.

## Data Availability

The following represents disclosure information provided by authors of this manuscript. All relationships are considered compensated. Relationships are self-held unless noted. I = Immediate Family Member, Inst = My Institution. Relationships may not relate to the subject matter of this manuscript. For more information about ASCO's conflict of interest policy, please refer to www.asco.org/rwc or ascopubs.org/jgo/site/misc/authors.html. **Honoraria:** Roche Mexico **Travel, Accommodations, Expenses:** Roche Mexico **Consulting or Advisory Role:** Roche Mexico, Novartis, Pfizer, AstraZeneca **Speakers' Bureau:** Roche, Myriad Genetics **Travel, Accommodations, Expenses:** Roche No other potential conflicts of interest were reported.
